# The Impact of Neighborhood‐Level Area Deprivation on Picture Naming Ability and Decline in Alzheimer’s Disease

**DOI:** 10.1002/alz.089756

**Published:** 2025-01-09

**Authors:** Tifani Biro, Roy Hamilton

**Affiliations:** ^1^ University of Pennsylvania, Philadelphia, PA USA

## Abstract

**Background:**

The cortex responsible for language processing is among the earliest areas affected by Alzheimer’s Disease (AD), manifesting speech and language abnormalities even before formal diagnosis. Language decline in AD is clinically significant, often parallel to a loss in basic daily living abilities. Research endeavors to detect AD early through automated speech analysis, emphasizing the need to comprehend factors influencing language disruption. Previous studies suggest that higher socioeconomic status (SES) may mitigate dementia and cognitive impairments. Our study explores the link between neighborhood‐level area deprivation and language decline in AD patients.

**Method:**

We utilized data from the University of Pennsylvania’s Integrated Neurodegenerative Disease Database (INDD). Inclusion criteria encompassed consensus diagnoses from multiple assessments for AD patients and individuals with normal cognition. Subjects' address information facilitated the derivation of State and National Area Deprivation Index (ADI) ranks based on 12‐digit Federal Information Processing System (FIPS) codes. Our analysis employed weighted linear mixed effects models to assess the impact of ADI Ranks on Boston Naming Task performance across patients with or without AD and among AD patients at various stages post‐symptom onset (2‐8 years).

**Result:**

Our analysis revealed an inverse relationship between ADI National and State Ranks and Boston Naming Scores, evident in both AD and non‐AD individuals. AD patients consistently demonstrated lower Boston Naming Task scores than their counterparts, with disparities widening as ADI Ranks increased. In AD patients, language decline correlated with disease progression, occurring earlier and intensifying with higher ADI National and State Ranks. These findings concur with prior research, underscoring the predictive utility of the Boston Naming Test for cognitive decline.

**Conclusion:**

Our study unveils the complex interplay between area deprivation and language decline in AD patients. Those residing in more deprived areas experienced accelerated language deterioration, highlighting the impact of socioeconomic disparities. These insights advance discussions on early AD detection and intervention strategies, underscoring environmental factors' role in disease trajectories and the potential for targeted interventions to enhance patient care.

